# Correlation between clinical and CBCT-based crestal soft tissue and bone parameters in posterior edentulous mandible

**DOI:** 10.6026/973206300212153

**Published:** 2025-07-31

**Authors:** Ajit Mishra, Arkaprava Banerjee, Savita Ghom, Anurag Bakshi, Anjusha Jadhav, Arpan Aash

**Affiliations:** 1 Department of Oral Medicine & Radiology, Maitri College of Dentistry and Research center, Anjora Durg, Chhattisgarh, India

**Keywords:** Crestal soft tissue, cone beam computed tomography (CBCT), cortical bone, cancellous density, thickness

## Abstract

Ideal bone considered for adequate implant stability is that with thick cortical bone surrounding a cancellous bone. This study, perhaps for the first time not only aimed to study the association among Crestal soft tissue thickness, Cortical bone thickness and Cancellous bone density but also to find gender related variations of the three parameters. The crestal soft tissue thickness was evaluated clinically and through CBCT. An increase in crestal soft tissue thickness is significantly associated with an improvement in thickness of cortical bone but cancellous bone density value had a little tendency to change.

## Background:

The science of medicine is a dynamic field that is always evolving. Our understanding of diagnosis, appropriate treatment and medication therapy is always growing as a result of research and clinical experiences [1]. The goal of present dentistry is to return the stomatognathic system to normal function, comfort, aesthetics, speech and health. Dental radiography has long been an interesting and useful diagnostic tool in the area of dentistry. One of the primary alternative therapy options for oral rehabilitation nowadays is the dental implant. Dental implants are becoming increasingly well-liked and widely accepted because, in addition to replacing lost teeth, they offer long-lasting restorations that don't affect speech or oral function or lower a patient's sense of self. This is due to on-going research into dental implant designs, materials, and processes as well as the expanding variety of imaging modalities. The sole kind of (non-surgical) bone testing needed for implant therapy is radiography [2]. It is impossible to overestimate the significance of pre-operative treatment planning for effective implant therapy. Diagnostic imaging is crucial in this situation [3]. Since the volume and quality of the surrounding bone play a major role in a dental implant's success, the degree of osseointegration becomes a crucial indicator of the early stability of the implant. Dental Implant successes rates are highly predictable. To prevent any issues during or after treatment, a comprehensive clinical and radiographic evaluation of the implant bed is crucial. One of the key elements affecting the treatment's outcome and prognosis is the early stability of the implant. Unfortunately, a thorough assessment of bone quality cannot be obtained from 2D pictures. Volumetric reconstructions of craniofacial structures are produced by CBCT. It can help with bone density measurement, which has a strong correlation with implant stability metrics. Consequently, CBCT scans can be used to forecast an implant's initial stability prior to implantation [4]. Since the volume and quality of the adjoining bone play a major role in a dental implant's success, the degree of osseointegration becomes a crucial indicator of the early stability of the implant [5]. One of the most sought-after results in implant dentistry is a stable peri-implant crestal bone. After a year of loading, a peri-implant crestal bone loss of less than 1.5 mm was considered a successful implant treatment. The aetiology of crestal bone loss is significantly influenced by the thickness of the vertical soft tissue [6]. The cortical bone plays a critical role in bone strength and is essential to comprehending how ageing affects bone structure. Numerous studies have shown a direct association among thickness of cortical bone and a number of age-related and gender-predisposed variables. It also has to do with microstructural alterations in the bone matrix, which serves as one of the primary supports for bony architecture and fracture resistance. A bone with dense cortical bone encircling a cancellous bone is ideal for sufficient implant stability [7]. To our knowledge till now there has been no clinical study to introspect into the inter-relationship between the three above mentioned variables. Therefore, it is of interest to correlate the Crestal Soft tissue thickness around the implant, cortical bone thickness and cancellous bone density. With this current preliminary detailed imbibition let's move into the identification of the possible correlation between these three factors indicated for primary implant stability.

## Materials and Methods:

A descriptive observational study for the assessment of clinically evaluated crestal soft tissue thickness; cortical bone thickness and cancellous bone density using CBCT in mandibular posterior edentulous region and the correlation between them were established after approval from the Institutional Ethical Committee. 40 patients meeting inclusion criteria were enrolled from the year 2023 -2025. Data was recorded and analysed using software package of IBM SPSS version 25 for Windows. A written informed consent from the entire patient was taken. Along with medical and dental history a proper menstrual history was taken. The patients having mandibular posterior edentulous spaces and indicated for implant placement were selected. Patients with pathologies, systemic/metabolic disease and pregnancy were excluded. Right and/or left edentulous posterior region is selected for each patient. Technical criteria set for each scan is as follows: FOV - 4 X 5, 7 X 7; Peak voltage - 85 kV Tube current - 10 mA Scan time - 15.4 seconds, 20 seconds. CBCT scanning is done of all the patients using CBCT machine PAPAYA 3D PLUS Combination Imaging System. The reconstructed images of each scan were evaluated in the axial, coronal and sagittal planes with cross section to arch every 0.5 mm spacing first using software Genoray THEIA Digital X - ray imaging solution, Version 1.0.0.13 provided with the PAPAYA 3D PLUS system. The crestal soft tissue thickness was evaluated clinically using endodontic k files with tissue stops number 20 which was then inserted through the soft tissue covering the edentulous area 2 minutes after surface anaesthesia using 15 % w/w Lidocaine Topical Anaesthetic spray. A scale was used to measure the length, which was then noted. Vertical cross-sectional views perpendicular to the alveolar ridge at the middle of each edentulous site were measured following CBCT imaging of the edentulous region. The alveolar crest's cortical bone thickness was assessed. At the buccal and lingual cortical plates, which are 5 mm apical to the alveolar crest, the thickness of the cortical bone was measured. Their average was taken into account. For the edentulous regions intended for implant implantation, greyscale values were measured. CBCT density recordings were made using THEIA Software's greyscale bone measuring tool in the buccolingual view of the axial plane. The entire length of the Implant/Mimic was measured and separated into four sections: the middle, apical, and coronal thirds, which comprise the compact most coronal 2 mm. Measurement was made parallel to the implant fixture from a distance of around 1 mm. A 2 mm line was drawn for each measurement, and greyscale values were noted at both ends. In each area, the mean was determined. The obtained data was statistically evaluated using Pearson's Correlation test at P less than 0.05.

## Results:

It was evident that correlation of crestal soft tissue thickness with cortical bone thickness had a strong positive correlation. It was also found that crestal soft tissue and cancellous bone density, the correlation obtained was a weak negative correlation. Finally, it was seen that with cortical bone thickness and cancellous bone density, the correlation obtained was a weak positive correlation (Table 1, 2 and 3 - see PDF).

## Discussion:

In our study, the mean crestal soft tissue thickness was 2.65 mm, exceeding the 2.5 mm threshold. Thin mucosal tissues can cause crestal bone loss after implant placement, according to a study by Xiaoxi *et al.* [8]. If the initial tissue thickness is less than 2.5 mm, bone loss of up to 1.45 mm is anticipated within the first year. The cortical bone is essential for primary implant stability and plays a major role in bone strength. The mean cortical bone thickness in our study was 2.245. The study by Ajai *et al.* [9] showed the mean cortical thickness to be 1.18 ± 0.48 which was similar to the values obtained in our study. Age-wise comparisons were not included in our study. High bone density is associated with both primary stability and a high percentage of implant success. Therefore, determining bone density is crucial before beginning any planned implant procedure [9]. The study by Manas *et al.* [10] showed an increase in the density to be highest postoperatively around implants at the coronal portion followed by the middle part and finally the apical part. Their mean was taken into consideration. The shape of the implant fixture, which is widest at the neck or the crest of the alveolar ridge and then taper gradually until it reaches the narrowest point and the least bone compressive point at the apex, may be related to the variation in the percentage increase in density. Some bone density studies such as the one by Elkhidir *et al.* showed that CBCT identified a postoperative increase in bone density which contributes to implant stability [11]. We also analysed the relationship between cortical bone thickness and crestal soft tissue thickness. According to our study's findings, the p value was 0.002 and the r value was 0.69 (Table 1 - see PDF). The association among the thickness of cortical bone and the thickness of crestal soft tissue was clearly positive and statistically significant (p≤0.05). This suggests that an increase in crestal soft tissue thickness is significantly associated with an increase in cortical bone thickness. Similar findings were reported by Chatvaratthana *et al.* [12] who demonstrated a significant correlation between crestal cortical bone thickness, crestal soft tissue thickness and implant stability quotient (ISQ). Numerous researches have looked at the connection between the thickness of the buccal cortical plate and the soft tissues of the face. One such study was conducted by Younes *et al.* [13], which examined the relationship between the thickness of the soft tissues in the pre-maxilla and the buccal bone. However, with increase in age the relation between crestal soft tissue thickness and the cortical bone thickness ratio alters. As mentioned earlier in older age group the residual ridge resorption is faster and thicker crestal soft tissue gets deposited [14]. Additionally, we evaluated the relationship between cancellous bone density and crestal soft tissue thickness. According to our study's findings, the p value was 0.69 and the r value was -0.06 (Table 2 - see PDF). It was clear that the connection between cancellous bone density and crestal soft tissue was weakly negative. This implied that the observed cancellous bone density value had a little tendency to change in the opposite direction as the thickness of the crestal soft tissue increased.

Statistical significance was not reached (p≥0.05). This outcome is most likely caused by a larger decrease of vertical bone height, which lowers the amount of accessible bone and eventually remodels the bony architecture to make it less dense. To the best of our knowledge, we were the first to examine the correlation between these two factors, and we discovered a weak negative correlation. Lastly, our study also attempted to determine the relationship between cancellous bone density and cortical bone thickness, yielding a p value of 0.29 and a r value of 0.16 (Table 3 - see PDF). It was clear that there was a weak positive association between cancellous bone density and cortical bone thickness. The statistical significance was low (p≥0.05). This implied that the measured value of cancellous bone grew slightly in tandem with an increase in cortical bone thickness. Our study had several limitations as with any other study like small sample size (n=40), considered only the mandibular posterior edentulous spaces specifically, post-operative changes after the implant changes were not taken in consideration as we concentrated on the pre surgical site examination only. Further studies with larger sample size, other jaw sites including the anterior mandible & the maxilla and post-operative site considerations are recommended. It was found that the measured value of cancellous bone grew slightly in tandem with an increase in cortical bone thickness. Many studies have evaluated the crestal soft tissue thickness through CBCT, though our study relied on clinical measurement measures. Using a straightforward clinical assessment of the soft tissue on top, we attempted to gain an indirect understanding of the quality and amount of the underlying bone. We anticipate that this will greatly assist doctors preparing for implant surgery with the pre-radiological evaluation of the underlying bone.

## Conclusion:

Our study suggested that an increase in crestal soft tissue thickness is significantly associated with an increase in cortical bone thickness. It was further clear that the observed cancellous bone density value had a little tendency to change in the opposite direction as the thickness of the crestal soft tissue increased. To the best of our knowledge, we were the first to examine the correlation between these two factors.
